# The influence of silane coupling agents on the properties of α-TCP-based ceramic bone substitutes for orthopaedic applications

**DOI:** 10.1039/d3ra06027f

**Published:** 2023-11-22

**Authors:** Piotr Pańtak, Joanna P. Czechowska, Aneta Zima

**Affiliations:** a Faculty of Materials Science and Ceramics, AGH University of Science and Technology Mickiewicza Av. 30 30-058 Kraków Poland pantak@agh.edu.pl azima@agh.edu.pl

## Abstract

Biomaterials based on α-TCP are highly recommended for medical applications due to their ability to bond chemically with bone tissue. However, in order to improve their physicochemical properties, modifications are needed. In this work, novel, hybrid α-TCP-based bone cements were developed and examinated. The influence of two different silane coupling agents (SCAs) – tetraethoxysilane (TEOS) and 3-glycidoxypropyl trimethoxysilane (GPTMS) on the properties of the final materials was investigated. Application of modifiers allowed us to obtain hybrid materials due to the presence of different bonds in their structure, for example between calcium phosphates and SCA molecules. The use of SCAs increased the compressive strength of the bone cements from 7.24 ± 0.35 MPa to 12.17 ± 0.48 MPa. Moreover, modification impacted the final setting time of the cements, reducing it from 11.0 to 6.5 minutes. The developed materials displayed bioactive potential in simulated body fluid. Presented findings demonstrate the beneficial influence of silane coupling agents on the properties of calcium phosphate-based bone substitutes and pave the way for their further *in vitro* and *in vivo* studies.

## Abstract 2

Novel, hybrid α-TCP-based bone cements containing silane coupling agents were developed and examined.

## Graphical abstract

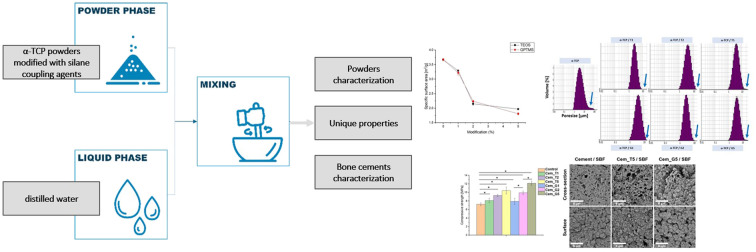

## 1. Introduction

Alpha tricalcium phosphate (α-TCP) due to its unique properties has been intensively studied as a promising material for bone tissue replacement and regeneration.^1–3^ Due to its chemical similarity to the inorganic part of bone tissue and self-setting properties α-TCP is commonly used as a main component of calcium phosphate-based cements (CPCs).^4^ CPCs possess excellent biocompatibility, osteoconductivity, and ability to bond with natural bone tissue.^5,6^ However, despite their numerous advantages, they still have some drawbacks, including their relatively low mechanical strength and fast resorption rates.^7,8^ To address these limitations, various modifications of powder and liquid phase of CPCs have been proposed.

α-TCP powder can be modified in different ways, for example through the incorporation of dopants, such as zinc, magnesium, or strontium, or by mixing with other calcium phosphate powders, such as β-TCP or hydroxyapatite.^7–10^ A common modification of CPCs formulation is application of different natural polysaccharides, which additionally provides injectability of cementitious pastes.^11–14^ Recently, it has been reported that calcium phosphates can be modified with silane coupling agents (SCAs), which are organosilicon compounds. Silane coupling agents can bond chemically to both the inorganic and organic surfaces, forming strong covalent bonds and improving the interfacial adhesion between different materials.^16,17^ SCAs have been widely used to modify the surface properties of various materials, including ceramics, polymers, and metals, to improve their performance in medical applications.^18–22^

Suppakarn *et al.*^23^ modified hydroxyapatite (HA) powders with different silane coupling agents, including γ-aminopropyl triethoxysilane (APES), methyl trimethoxysilane (MTMS), and γ-glycidoxypropyl trimethoxysilane (GPMS) to obtain HA/polypropylene composites. The results demonstrated that the silane treatment enhanced the interaction between the HA and PP, resulting in an increase in the stiffness of the composite material. The γ-aminopropyl triethoxysilane (APES) was found to be the most effective silane coupling agent for this application. Ji *et al.*^24^ developed hydroxyapatite-based scaffolds by modifying the surface of HA with different silane coupling agents: 3-methacryloxypropyltrimethoxysilane, 3-aminopropyltrimethoxysilane and carboxyethylsilanetriol sodium salt. The using of SCAs modifiers enhanced mechanical strength up to 30 MPa, which was explained by the presence of electrostatic bonds between components. Furthermore, the functionalized silane-coated HAp scaffolds displayed excellent biocompatibility. Ghorbani *et al.*^25^ incorporated 3-glycidoxypropyl trimethoxysilane (GPTMS) as a bioactive inorganic crosslinker in chitosan-polyvinyl alcohol scaffolds which led to the improvement of mechanical strength, water uptake, and biodegradation properties. It has been demonstrated that an increase in GPTMS content enhanced the compressive strength of samples, while reducing water uptake and biodegradation of materials. What is more obtained composites possessed excellent biological properties confirmed by different assays. Another common SCA is tetraethoxysilane (TEOS) which is used in the development of biomaterials due to its ability to form biocompatible silica coatings, enhancing their biocompatibility and stability within the cellular environment. Tallia *et al.*^26^ developed promising SiO_2_–CaO/PTHF/PCL-diCOOH hybrid material with improved mechanical properties, enhanced dissolution behaviour, and cell attachment capability. Fuh *et al.*^27^ described novel TEOS treatment method that enhances micro-porosity, reduces sintering shrinkage, and improves biodegradation in hydroxyapatite (HA) scaffolds, making it a promising technique for HA manufacturing.

Despite the potential advantages of using silane coupling agents to modify calcium phosphates powders, to the best of our knowledge, no modification of α-TCP has been carried out yet. The simultaneous hydrolysis of α-TCP and silane coupling agent may potentially lead to the formation of a novel α-TCP-based hybrid materials with improved properties due to presence of additional bonds between the components. The SCAs during hydrolysis can react with the hydroxyl groups on the surface of calcium phosphates, forming covalent bonds and modifying the properties of material. Such interactions, have been described previously in other biomaterials.^28,29^ In this work, two different SCAs were examined as potential moderators of interactions between materials components. We believe that the use of SCAs can lead to favourable properties of hybrid, α-TCP-based bone cements due to the formation of new bonds in the materials' structure.

The aim of this study was to develop bone cements on the basis of α-tricalcium phosphate and investigate the influence of tetraethoxysilane (TEOS) and 3-glycidoxypropyl trimethoxysilane (GPTMS) on their physicochemical and biological properties.

## 2. Materials and methods

### 2.1. Synthesis of α-TCP

The initial α-TCP powder was synthesized by the wet chemical method described previously.^30^ As reagents, Ca(OH)_2_ (≥99.5%, POCH, Gliwice, Poland) and H_3_PO_4_ (85.0%, POCH, Gliwice, Poland) were applied. In brief, the α-TCP precipitate after synthesis was dried, sintered above 1250 °C (5 h), ground in an attritor mill (3 h), and sieved below 63 μm.

### 2.2. Modification of α-TCP

The α-TCP powder was modified with 1, 2 and 5 wt% TEOS (T) (≥99.5%, Sigma-Aldrich, St. Louis, MO, USA) or GPTMS (G) (≥98.0%, Sigma-Aldrich, St. Louis, MO, USA) (Table 1). To modify the initial powders, solutions of the appropriate silane coupling agents were prepared using anhydrous ethanol as a solvent (99.8%, POCH, Gliwice, Poland). The alcoholic solvent was used to avoid hydrolysis of the calcium phosphate powders and SCAs. After adding the powders to the SCA solutions, they were stirred on a magnetic stirrer for 4 hours. The resulting precipitates were subjected to aging (1 h) and subsequently silanised by thermal treatment at 115 °C (4 h). Prior to preparing the samples, the powders were sieved through a sieve below 63 μm.

**Table 1:** Composition of the initial powders

Label	Description
α-TCP	Non-modified α-TCP powder
α-TCP/T1	α-TCP powder modified with 1 wt% of TEOS
α-TCP/T2	α-TCP powder modified with 2 wt% of TEOS
α-TCP/T5	α-TCP powder modified with 5 wt% of TEOS
α-TCP/G1	α-TCP powder modified with 1 wt% of GPTMS
α-TCP/G2	α-TCP powder modified with 2 wt% of GPTMS
α-TCP/G5	α-TCP powder modified with 5 wt% of GPTMS

### 2.3. Bone cements preparation

Seven types of materials were prepared by mixing the solid phase, *i.e.*, non-modified and SCA-modified α-TCP with the liquid phase. The initial composition of the prepared cements, as well as a liquid to powder ratio (L/P), are presented in Table 2.

**Table 2:** Composition of the bone cements

Label	Powder phase	Liquid phase	L/P [g g^−1^]
Cement	α-TCP	Distilled water	0.45
Cem_T1	α-TCP/T1		
Cem_T2	α-TCP/T2		
Cem_T5	α-TCP/T5		
Cem_G1	α-TCP/G1		
Cem_G2	α-TCP/G2		
Cem_G5	α-TCP/G5		

### 2.4. Methods

#### 2.4.1. Specific surface area

The specific surface area (SSA) of the initial α-TCP and SCAs-modified α-TCP powders was determined by the BET (Brunauer–Emmett–Teller) method using accelerated surface area and porosimetry system ASAP 2010 (Micromeritics, Norcross, GA, USA).

#### 2.4.2. Powders size distribution and zeta potential

The size distribution of the initial α-TCP and SCAs-modified α-TCP powders as well as zeta potential was based on Brownian motion and the Dynamic Light Scattering (DLS) technique and combination of electrophoresis and the Laser Doppler Velocimetry technique using system Zetasizer Nano-ZS (Malvern Panalytical, Malvern, UK).

#### 2.4.3. Chemical and phase composition

The X-ray fluorescence method (XRF) was applied to check the chemical composition of the initial powders (WDXRF Axios Max, PANalytical, Malvern, UK). The X-ray diffraction (XRD) analysis was performed using Cu Kα radiation (1.54 Å) at 30 kV and 10 mA. The analysis was conducted in the 2*θ* range of 10–60° at 0.04 intervals with a scanning speed of 2.5° min^−1^ using D2 Phaser diffractometer (Bruker, Ballerica, MA, USA). The obtained diffractograms were compared with the International Centre for Diffraction Data α-TCP (00-009-0348) and hydroxyapatite (HA; 01-076-0694) to identify the crystalline phases. TOPAS software (Bruker, Ballerica, MA, USA) was used for phase quantification based on Rietveld refinement. All measurements were performed in triplicate. The mean ± standard deviation (SD) was used to present the results.

FT-IR and Raman spectroscopy methods were used for the characterization of obtained non-modified and modified powders, as well as the hardened bone cements. FTIR investigations of materials were conducted on a BioRad FTS 6000 spectrometer (Bruker, Ballerica, MA, USA) with the Raman attachment (NdYAG laser excitation line of 1064 nm) in the wavenumber range of 3800–200 cm^−1^. The transmission technique was used and the samples were prepared as standard KBr pellets. This methodology was used to obtain an accurate and detailed understanding of the chemical and structural properties of the materials, which is crucial for determining their suitability for specific biomedical applications.

#### 2.4.4. Setting times

The setting times were determined in accordance with the ASTM C266-20 standard, using Gilmore Needles (Humbold MFG Co., Norridge, IL, USA).^31^ The Gillmore Apparatus, consisting of two steel-weighted needles, was used for this purpose. The initial setting needle weighed 113 g and had a diameter of 2.12 mm, while the final setting needle weighed 453.6 g and had a diameter of 1.06 mm. To determine the setting times, the cementitious pastes were placed in a special form measuring 8 mm × 10 mm × 5 mm, and the needle of the apparatus was lightly applied to its surface. The setting time was measured as the point at which the needle penetrated the surface without leaving a complete circular mark. All experiments were carried out at room temperature (22 ± 1 °C), and the results were presented as the average of three measurements, along with their corresponding standard deviations (SD).

#### 2.4.5. Mechanical strength

To determine the compressive strength of the materials, cylindrical samples with a height of 12 mm and diameter of 6 mm were tested using a universal material testing machine (Instron 3345, Norwood, MA, USA) at a crosshead displacement rate of 1 mm min^−1^. The compressive strength results were expressed as the mean value ± the standard deviation (SD) of twenty-fold determinations. To determine if there were any statistically significant differences between the materials, a one-way ANOVA followed by post hoc Tukey's Honest Significant Difference (HSD) test was applied with a significance level of *p* < 0.01.

#### 2.4.6. Microstructure

For microstructure observations of the fractured samples, scanning electron microscopy (SEM) was employed using a PhenomPure instrument (Thermo Fisher Scientific, Waltham, MA, USA). To evaluate the bioactive potential of the materials, their surfaces were assessed after 7 days of incubation in simulated body fluid (SBF) at 37 °C. Before examination, the samples were coated with a thin layer of gold film using a low deposition rate to prevent any charge build-up and to enhance the imaging resolution.

#### 2.4.7. *In vitro* bioactivity and chemical stability

To evaluate the chemical stability and bioactivity of the bone cements, they were incubated in distilled water or simulated body fluid (SBF) prepared according to Kokubo's protocol.^32^ Cylindrical samples were placed in containers with 20 mL SBF or water and stored at 37 °C. The chemical stability of the materials was determined by measuring the pH and ionic conductivity of the solutions around incubated samples at various time intervals. Measurements were taken using a Seven Compact Duo pH/conductometer (Mettler Toledo, Columbus, OH, USA). Results were expressed as the average value of three measurements ± standard deviation (SD). After immersion, the samples were removed from solution, rinsed with distilled water, and dried at temperatures below 40 °C. To confirm the bioactive potential, the surfaces of the samples were observed using SEM.

## 3. Results and discussion

### 3.1. Specific surface area

**Fig. 1:**
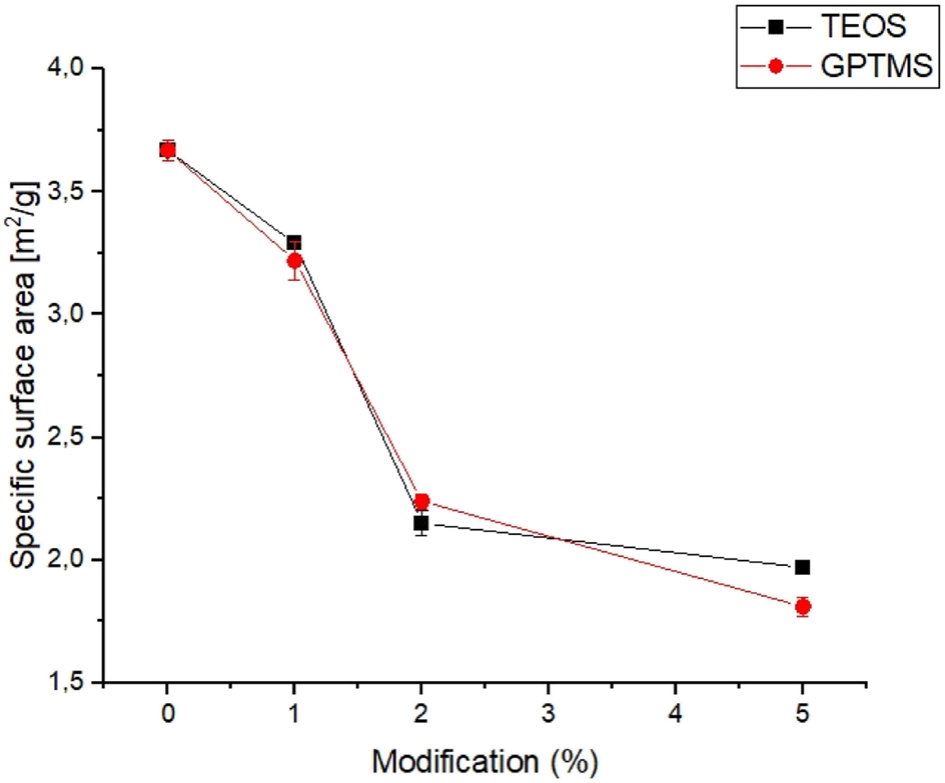
Specific surface area *vs.* amount of used silane coupling agent.

The specific surface area is an important parameter for characterizing the performance of catalysts, adsorbents, and other porous materials, as it can affect the reactivity, selectivity, and stability.^33,34^ The specific surface area of obtained powders is shown in Fig. 1.

The specific surface area of the obtained calcium phosphate powders was in the range of 1.81 ± 0.04 to 3.67 ± 0.04 and decreased with increasing amounts of the silane coupling agent. The highest value of specific surface area was noticed for non-modified α-TCP powder. From the results obtained, it can be seen that the specific surface area of the powders decreases with use and increasing amount of silane coupling agent. Generally, the specific surface area of α-TCP ranged from 2.0 to 11.0 g m^−2^ and depends on many factors, such as synthesis, processing routes and presence of modifiers.^35,36^ The decreasing value of the specific surface area of silane coupling agent-modified powders is due to the confinement of micropores in the powder grains with the silane coupling agents. The silane coupling agents form a thin layer on the surface of the grains, which reduce the overall surface area of calcium phosphate powders. Similar results were obtained by Sonn *et al.*^37^ and Chuang *et al.*,^38^ who modified the surface of silica nanoparticles with various coupling agents. They have also shown that the specific surface area of nanoparticles decreased with increasing concentration of the silane coupling agents due to reduction of micropores in the powders.

### 3.2. Powders size distribution and zeta potential

**Fig. 2:**
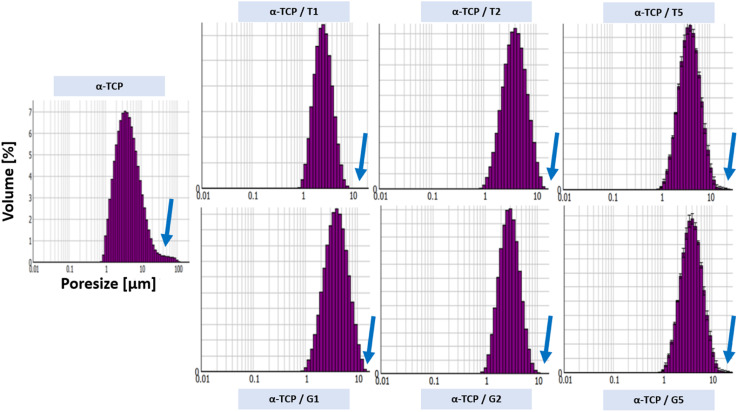
Powders size distribution of non-modified and modified α-TCP powders.

The powder size distribution and zeta potential are crucial parameters in powder technology, especially for calcium phosphates, as they can affect the properties and performance of the powders. Optimal values of this parameters depend on specific requirements. The powder size distribution (Fig. 2) and zeta potential (Table 3) of obtained powders are shown below.

**Table 3:** Zeta potential of obtained powders

Powder label	Zeta potential [mV]
α-TCP	−4.65 ± 0.3
α-TCP/T1	−9.67 ± 0.5
α-TCP/T2	−12.72 ± 0.3
α-TCP/T5	−16.31 ± 0.7
α-TCP/G1	−18.3 ± 1.5
α-TCP/G2	−21.2 ± 1.0
α-TCP/G5	−24.4 ± 0.9

It was observed that the surface modification of the α-TCP powders with both silane coupling agents changed a powders size distribution. For non-modified α-TCP pore size were in the range of approximately 1.0 to 100.0 μm. However, the SCA modification caused change of powders size distribution to the range between ∼1.0 to 10.0 μm. When silane coupling agents are used to modify the surface of the powder particles, they can form a monolayer or multilayer made of organic molecules, which can reduce the surface energy and enhance the dispersibility of the particles in the surrounding medium.^39^ Additionally, as TEOS and GPTMS has a hydrophobic functional groups, what can reduce the water affinity of the particle surface and promote the formation of smaller agglomerates.^40–42^ The differences in zeta potential measurements also were observed. The non-modified powder possessed the least electro-negative zeta potential (*i.e.* −4.65 ± 0.3 mV). However, the surface modification caused changes in zeta potential values. As in the case of powders size distribution the changes in zeta potential values were caused by the presence of layer of SCA molecules, which can reduce the electrostatic repulsion between CaPs particles and decrease the zeta potential^43^ (up to −24.4 ± 0.9 mV for α-TCP/G5).

### 3.3. Chemical and phase composition

**Fig. 3:**
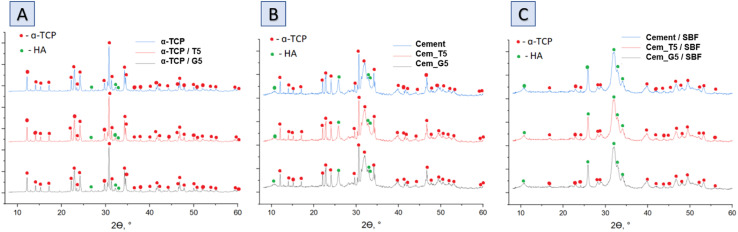
Diffractograms of obtained materials: powders (A), bone cements after setting and hardening in air for 7 days (B) and bone cements incubated in SBF for 7 days (C).

The XRD analysis revealed that the initial α-tricalcium phosphate and silane coupling agents modified α-TCP powders composed mainly of α-TCP (97–98 wt%) and a small amount of hydroxyapatite phase (2–3 wt%) (Fig. 3A). As expected, the modification of the powders *via* SCAs in anhydrous medium did not result in hydrolysis of α-TCP to non-stoichiometric hydroxyapatite. Additionally, by using silane coupling agents to modify the surface of the powders, silicon ions were introduced. The applied method of modification allowed for introduction to α-tricalcium phosphate, and no other crystalline phases containing silicon were observed. The X-ray fluorescence method confirmed the presence of silicon in all modified powders and in amount of 0.035 ± 0.001 wt% for non-modified powder. As expected, the silicon content increased with the increasing amount of SCAs used and was 0.218 ± 0.004 wt% and 0.280 ± 0.002 wt% for powders modified with 5 wt% of TEOS and GPTMS respectively. Presence of silicon may improve the biological properties of the final material, such as osteoconductivity.^44^ The modification allowed the powder to be enriched with silicon ions after α-TCP synthesis. Overwhelming majority of scientific publications describe the introduction of silicon ions into calcium phosphate-based materials at the synthesis stage.^45^

The diffractograms of bone cements after setting and hardening in air for 7 days (Fig. 3B and Table 4), as well as cements incubated in SBF for 7 days (Fig. 3C and Table 3), revealed two crystalline phases, *i.e.*, α-TCP and hydroxyapatite, regardless the amount of the silane coupling agents. It has been demonstrated that in the simulated body fluid, the α-TCP phase is thermodynamically metastable and almost completely hydrolyses to nonstoichiometric hydroxyapatite. This transformation is accompanied by a change in the chemical composition and crystallographic structure of the material, which can affect its properties and behaviour in biological and biomedical applications.^46^

**Table 4:** Phase composition of tested bone cements

Material label	After 7 days of setting and hardening in air (22 ± 1 °C)		After 7 days of incubation in SBF (37 ± 1 °C)	
	α-TCP	Hydroxyapatite	α-TCP	Hydroxyapatite
Cement	90.7 ± 0.5	9.3 ± 0.5	10.4 ± 1.0	89.6 ± 1.0
Cem_T5	87.9 ± 1.0	12.1 ± 1.0	6.4 ± 1.0	93.6 ± 1.0
Cem_G5	85.4 ± 1.0	14.6 ± 1.0	5.8 ± 0.5	94.2 ± 0.5

**Fig. 4:**
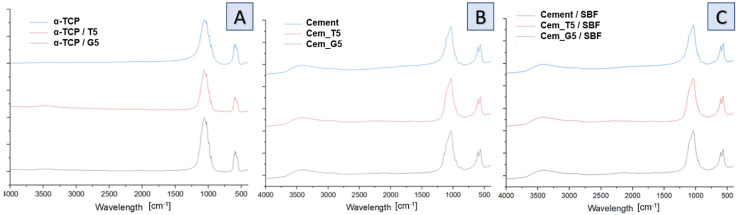
FT-IR spectra of obtained materials: powders (A), bone cements after setting and hardening in air for 7 days (B) and bone cements incubated in SBF for 7 days (C).

Infrared spectra of initial powders (Fig. 4A), cements after setting and hardening (Fig. 4B) as well as cements after 7 days of incubation in simulated body fluid (Fig. 4C) revealed the presence of several characteristic bands, which provided valuable insights into the composition and structure of the materials. Similar results were obtained for all SCAs concentrations, and no significant differences were observed for the various amount of silane coupling agents. Specifically, bands at around 565 and 605 cm^−1^ were observed and assigned to triply degenerate υ4 P–O–P bending modes. Additionally, a strong doublet near 600 and 670 cm^−1^ was connected to SO_4_ bending vibrations (υ4). Interestingly, the coincidence of SO_4_ and PO_4_ bending vibration bands was observed at around 603 cm^−1^. The other important bands were observed at 1060 cm^−1^ and around 1040 cm^−1^, which were assigned to υ3 non-symmetric stretching modes of P–O and are characteristic of calcium phosphates. Furthermore, a band at 962 cm^−1^ was observed, which was associated with the υ1 symmetric stretching vibration of P–O bands. In addition, a wide band around 3500 cm^−1^ indicates the presence of residual water in the studied samples. Low concentration of silane coupling agents in the cements, as well as an overlapping of bands, may explain the lack of visible peaks of Si–O–Si, Si–OH, Si–C and C–H bands assigned to SCAs.^47^ In addition bone cements after setting and hardening in air, as well as after incubation in SBF possessed the absorption bands arising from HPO_4_^2−^. The weak band at around 870 cm^−1^ assigned as P–OH stretch of HPO_4_^2−^ to calcium deficient materials. Thus, obtained data confirmed the presence of calcium-deficient hydroxyapatite (CDHA) in material at the same time shows its bioactive potential due to hydrolysis of α-TCP to non-stoichiometric hydroxyapatite.

**Fig. 5:**
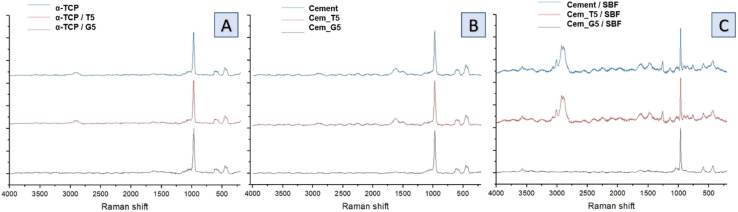
Raman spectra of obtained materials: non-modified powder and cements (A), TEOS-modified powder and cements (B) and GPTMS-modified powder and cements (C).

Raman spectra of non-modified powder and cements (Fig. 5A), TEOS-modified powder and cements (Fig. 5B) as well as TEOS-modified powder and cements (Fig. 5C) revealed the presence of bands characteristic for calcium phosphates, as well as band unique for TEOS and GPTMS. Specifically, CPCs based on α-TCP are characterised by bands around 450 and 560 cm^−1^ attributed to the symmetric stretching and bending modes of the Ca–O bonds. This bands may shift slightly depending on the conditions. Additionally, phosphate groups at around 960, 1005, and 1080 cm^−1^ are attributed to the symmetric stretching, bending, and asymmetric stretching modes, respectively. The small amounts of carbonate ions, which are confirmed by bands at around 1060 and 1415 cm^−1^. These bands are attributed to the symmetric stretching and bending modes of the carbonate ion, especially for cements after its incubation in SBF. The presence of remaining water in CPCs was also confirmed by the presence of characteristic bands at around 3300 and 3500 cm^−1^. In addition to the bands characteristic for calcium phosphates, bands characteristic for silane coupling agents also were observed. TEOS exhibits a Si–O–Si band at around 1100 cm^−1^, which is attributed to the symmetric stretching mode of the Si–O–Si bond. It also exhibits band at around 2940 cm^−1^, attributed to the stretching mode of the Si–CH_3_ bond and band around 1250 cm^−1^ is assigned to the stretching mode of the Si–O–CH_3_ bond in TEOS. The presence of Si–OH groups in TEOS were confirmed by bands around 3600–3700 cm^−1^, which is attributed to their stretching mode. On the other hand, GPTMS presence in obtained materials was confirmed by the presence of Raman band at around 910 cm^−1^, attributed to the stretching mode of the CH_2_–O bond. This characteristic bond of epoxide groups is more visible for incubated materials. Similarly, to TEOS, GPTMS also exhibits bands assigned to a Si–O–Si (∼1100 cm^−1^), Si–CH_3_ (∼2940 cm^−1^) and Si–O–CH_2_ (∼1250 cm^−1^).

During the simultaneous hydrolysis of α-TCP and silane coupling agents several potential new bonds can be formed between the coupling agents and the surface of the α-TCP particles. This includes the Si–O bond, which can be formed when the silanol group of the TEOS or GPTMS molecule reacts with the groups on the surface of CaPs grains. The stretching vibration of the Si–O bond typically appears between 1000–1200 cm^−1^, while the stretching vibration of the –OH appears around 3500 cm^−1^. The formation of the Si–O bond results in a shift of the Si–O stretching vibration. Ribeiro *et al.*^48^ demonstrated interaction between ultra-fine calcium carbonate and natural rubber by applying a superficial treatment to the calcium carbonate which provides materials with improved properties. They observed the similar bonds between coupling agents and calcium carbonate groups. Bi *et al.*^49^ investigated the effects of trimethoxysilanes on the properties of CaCO_3_-based composites. They confirmed the presence of chemical interaction between components, which allow to improve the tensile strength and abrasion resistance of the materials. Moreover, Bi *et al.* stated that the silanol groups of two coupling agents react with each other and form a covalent bond Si–O–Si, creating the hybrid material. Purcar *et al.*^50^ synthesized hybrid nanomaterials based on zinc oxide *via* the sol–gel method, using different silane coupling agents. They confirmed the presence of Si–O–Si bands in the materials and showed their positive influence on the materials' properties. Furthermore, the presence of Si–O–P bond at 1000 and 1200 cm^−1^ can confirm the interactions between the silanol group on the TEOS or GPTMS molecule and the phosphate groups, forming a covalent bond between the silicon atom of the coupling agent and the oxygen atom of the phosphate group. These bonds can enhance the further properties of the materials, such as mechanical strength and bioactivity. In addition, the introduction of coupling agents to cements composition may allow for its further modification using polymers with different functional groups that can also react with SCAs.

### 3.4. Setting times

Setting time is critical parameter for calcium phosphate cements because it affects their handling, injectability, and mechanical properties.^51^ The setting times of the obtained bone cements depended on the amount of used silane coupling agent and varied between 3.5 and 6.5 min (initial setting time) and 6.5 and 11.5 min (final setting time) (Table 5).

**Table 5:** Setting times of developed materials

Material	Initial setting time (*T*_i_) [min]	Final setting time (*T*_f_) [min]
Control	6.5 ± 0.5	11.0 ± 1.0
T1	6.5 ± 0.5	11.5 ± 0.5
T2	5.0 ± 1.0	9.0 ± 0.5
T5	4.0 ± 0.5	7.5 ± 1.0
G1	5.0 ± 0.5	9.0 ± 1.0
G2	4.0 ± 1.0	7.5 ± 0.5
G5	3.5 ± 1.0	6.5 ± 0.5

The results showed that the addition of silane coupling agent used for calcium phosphate modification decreased both, the initial and final setting time. The process responsible for setting and hardening of calcium phosphate cements involves α-TCP hydrolysis. When α-TCP reacts with water, it forms calcium-deficient apatite, as described by the following eqn (1):^52,53^

1 3Ca_3_(PO_4_)_2_ + H_2_O → Ca_9_(PO_4_)_5_(HPO_4_)OH

Several factors can affect the setting reaction of bone cements, such as the amount of highly reactive α-TCP phase in the material, such as: the presence of setting accelerators in the liquid phase, the type and amount of polymeric additive in the liquid or powder phase, and α-TCP powder modifications.^54^ Results of our studies revealed that the presence of silane coupling agents also influence the setting time values. It seems that SCAs accelerate the setting process of α-TCP-based bone cements. Previous studies have demonstrated that the incorporation of silicon into calcium phosphate cement alters its properties in comparison to the non-modified cements. For example, Czechowska *et al.*^45^ developed calcium phosphate-based bone fillers based on silicon doped α-TCP with hybrid, gold-modified granules. Their study showed a shortening of both initial and final setting times for silicon-containing materials. The study by Wei *et al.*^55^ confirmed a higher solubility of silicon-modified α-TCP, additionally Mestres *et al.*^56^ found a faster hydrolysis of silicon-modified α-TCP in comparison with non-modified powders. All this finding corresponds with the results obtained during experiments. Although the measured setting times were reduced due to the use of a silane coupling agent, their values remained in the range suitable for clinical application (4–8 min for initial setting, up to 15 min for final setting time^46^).

### 3.5. Mechanical strength

**Fig. 6:**
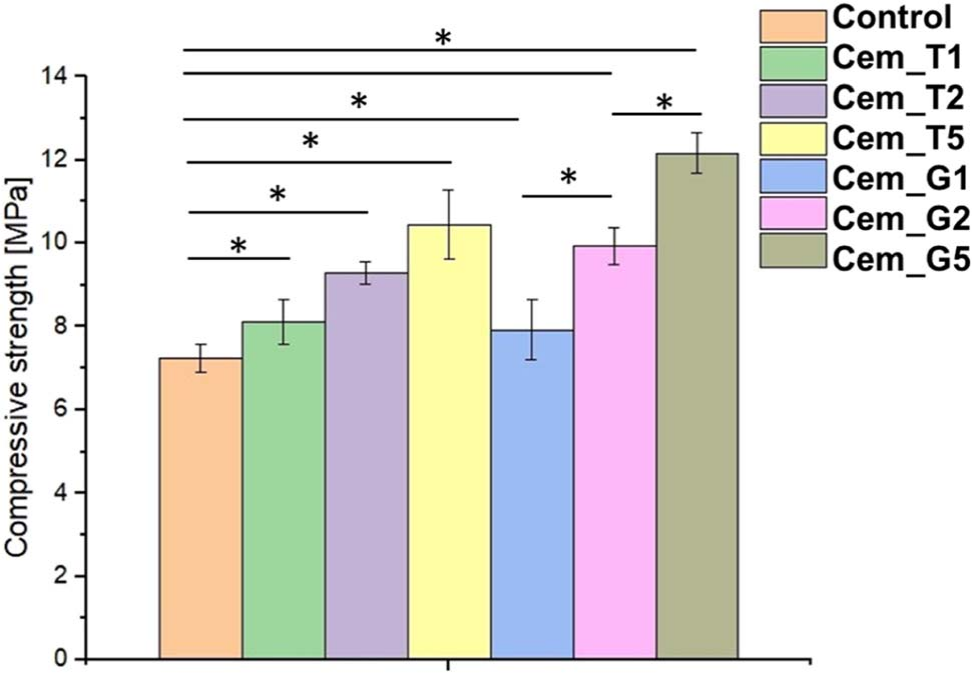
Compressive strength of obtained bone cements after 7 days of setting and hardening (* – statistically significant difference, *p* < 0.01).

The mechanical strength of calcium phosphate-based bone cements is important parameter, because it determines the ability of the cement to withstand the mechanical forces and stresses in the bone environment, ensuring successful bone repair and regeneration. The compressive strength of obtained biomaterials ranged from 7.24 ± 0.35 MPa to 12.17 ± 0.48 MPa (Fig. 6). The results of the mechanical tests revealed that the presence of SCAs modifier positively affected the compressive strength of the bone cements. The highest values were obtained for material G5 (with 5 wt% of GPTMS). Based on the results of a one-way ANOVA and a subsequent Tukey HSD post-hoc analysis, it can be concluded that the observed differences between the control and SACs modified materials were statistically significant.

The silane coupling agents as modifiers of α-TCP may provide the mechanical improvement of obtained bone cements due to formation of additional bonds in materials' structure during the simultaneous hydrolysis of α-TCP and SCAs. The increase in the mechanical strength of the developed cements is possibly related to the chemical interactions between their components and formation of additional Si–O–Si, Si–O, or Si–O–P bonds what led to creation of hybrid-type materials. Similar, positive influence of using silane coupling agents on mechanical behaviour of different biomaterials where previously described in the literature. In the study by Ma *et al.*^57^ the trimethoxysilane was used as a modifier of hydroxyapatite/polyether ether ketone (PEEK) composites to obtain materials with improved mechanical strength. Furthermore, Ma *et al.* shoved promising results from *in vivo* studies. Vaz *et al.*^58^ shoved beneficial application of different coupling agents to obtain starch/ethylene-vinyl alcohol copolymer/hydroxyapatite composites characterised by increased mechanical resistance due to improved adhesion between material components. Harper *et al.*^59^ presented the beneficial properties of hydroxyapatite loaded acrylic bone cements. By modification of hydroxyapatite by using 3-trimethoxysilylpropylmethacrylate authors obtained materials characterised by increased mechanical parameters. It should be noted that the compressive strength of cancellous bone ranges from approximately 4 to 12 MPa.^60^ Thus, the hybrid-type, SACs modified materials developed in our study possessed mechanical strength suitable for implantation in non-load or low-load bearing locations.

### 3.6. Microstructure

To evaluate the potential of the developed materials as a bone tissue substitutes and detect any microstructural defects, their microstructure was examined. The initial powders and obtained biomaterials were observed using scanning electron microscopy (SEM) after setting and hardening in air, as well as after incubation in SBF.

**Fig. 7:**
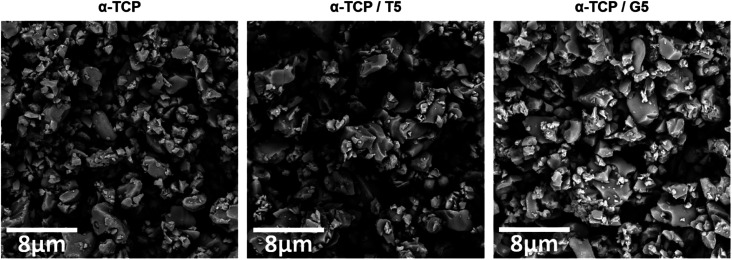
Morphology of initial α-TCP powders (magnification: 100 00×).

SEM observations revealed that the morphology of powder grains of non-modified and TEOS or GPTMS modified α-TCP did not differ significantly (Fig. 7).

**Fig. 8:**
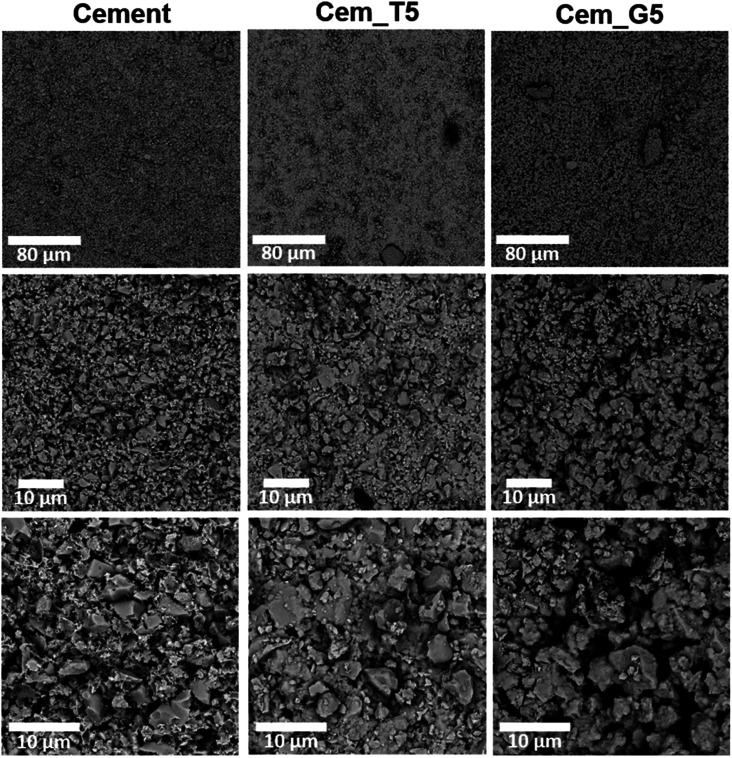
Microstructure of obtained materials after seven days of setting and hardening in air (cross-sections, magnifications: 1000, 5000, 100 00×).

The set and hardened materials possessed compact, homogenous microstructure formed by cementitious matrix with visible micropores (Fig. 8). Regardless the amount of silane coupling agent no differences in materials' microstructure were observed. Similar microstructures of α-TCP-based bone cements can be found in other studies.^61^

**Fig. 9:**
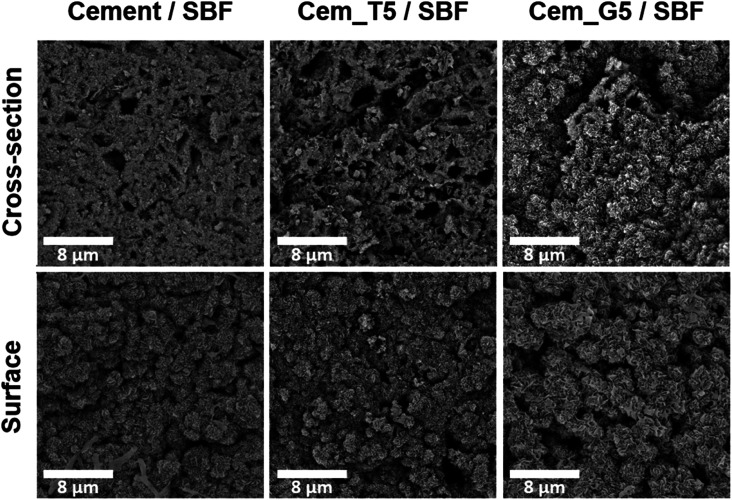
Microstructure of obtained materials after 7 days incubation in SBF (magnification: 100 00×).

After 7 days of incubation in simulated body fluid (SBF) all prepared materials were completely covered by plate-like apatitic structure confirmed their bioactivity *in vitro* according to Kokubo and Takadama's criteria (Fig. 9).^62^ Furthermore, the microstructure analysis after incubation showed that the developed materials retained their bioactive potential despite the using silane coupling agents as a modifier of α-TCP powders.

### 3.7. *In vitro* bioactivity and chemical stability

**Fig. 10:**
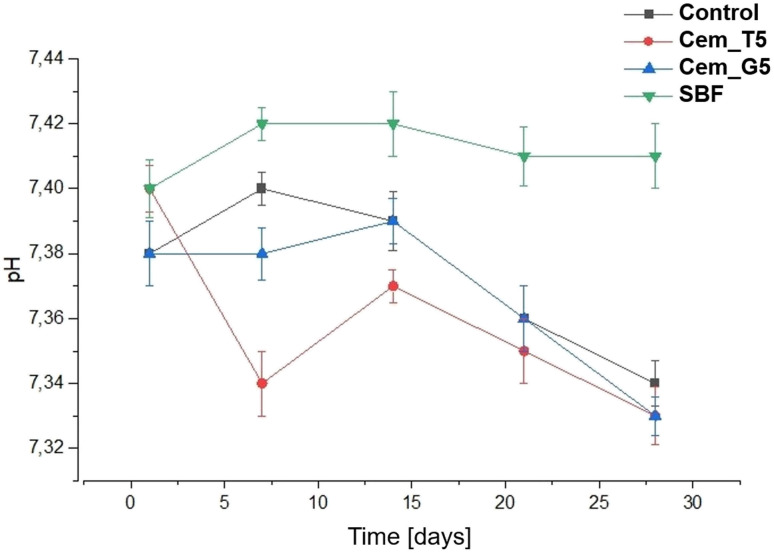
pH *versus* bone cements' incubation time in SBF.

The chemical stability of implantable biomaterials is crucial parameter in determining their potential clinical application. The pH changes of the SBF during the samples' immersion are illustrated in Fig. 10.

The pH changes of SBF around incubated samples remained close to the physiological values and ranged from 7.34 to 7.40. The addition of TEOS and GPTMS only slightly influenced the solution's pH values. Similar pH values of incubated calcium phosphate-based bone substitutes were observed elsewhere.^15^

**Fig. 11:**
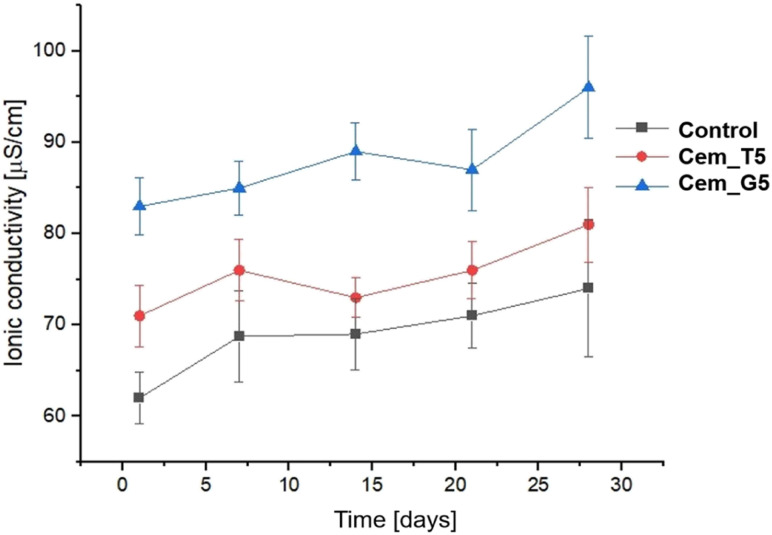
Ionic conductivity *versus* bone cements' incubation time in distilled water.

Ionic conductivity during the incubation in distilled water of the control material was in the range of ∼72–81 μS cm^−1^. A CPCs modifiers caused slightly increase in ionic conductivity to the range of ∼73–87 μS cm^−1^ for TEOS, and to the range of ∼84–96 for GPTMS (Fig. 11). This phenomenon can be explained by a higher degradation rate silane coupling agents and the releasing of ion during its continuous hydrolysis in aqueous solutions.^63^ Ionic conductivity of all the obtained materials is in the range typical for α-TCP-based bone cements. It can therefore be concluded that the modification with silane coupling agents did not significantly affect the degradation rate of bone cements.

The bioactive potential of obtained materials was estimated through its incubation in SBF.^62^ The SEM observations show that the evenly distributed, bone-like apatite layer was present on the sample's surfaces and cross-sections after seven days of incubation in SBF at 37 °C (Fig. 9). The presence of apatite forms indirectly indicates the high bioactive potential of the obtained materials.

## 4. Conclusions

The positive effect of silane coupling agents on the mechanical behaviour of various biomaterials has been previously described, but there is little information about the effect of SACs on the properties of calcium phosphate bone cements. In this study, we developed and examined the novel, hybrid-type calcium phosphate bone cements based on α-TCP powders modified with silane coupling agents (SCAs) – tetraethoxysilane (TEOS) or 3-glycidoxypropyl trimethoxysilane (GPTMS). Developed materials, due to their hybrid nature, combine the excellent biological properties and improved physicochemical properties. Application of SACs allowed to increase the compressive strength of the composites from 7.24 ± 0.35 MPa to 12.17 ± 0.48 MPa. The strengthening effect may be explained by the chemical interactions between the components during the simultaneous hydrolysis of α-TCP and silane coupling agents. It has been shown that SCAs during hydrolysis can react with the hydroxyl groups on the surface of calcium phosphates, forming covalent bonds (*e.g.* Si–O–Si, or Si–O–P). In addition, the silanol groups have the tendency to form hydrogen bonding between each other. Furthermore, the results of our studies revealed that modification of α-TCP by SCAs influenced the setting process of bone cements. The decrease of final setting time from 11.0 to 6.5 minutes was observed. SEM observations showed that all of the developed bone substitutes have a homogenous microstructure. All of the studied materials possessed a bioactive potential, proven in *in vitro* studies in simulated body fluid. By using the silane coupling agents, we developed materials containing silicon, which may positively impact their osteoconductivity. Based on the obtained results, it can be concluded that the most favourable enhancement of the physicochemical properties of the developed materials was achieved by the modification of α-TCP with 5 wt% GPTMS.

All presented findings confirm the beneficial influence of silane coupling agents on the properties of calcium phosphate-based bone cements and pave the way to further *in vitro* and *in vivo* studies. Developed hybrid-type materials may be further modified, by introducing natural polymers, what should lead to creation of chemically coupled hybrid-type bone cements.

## Conflicts of interest

There are no conflicts to declare.

## Supplementary Material
